# The Relationship Between CTLA-4 (-318 C/T) Polymorphism and Urothelial Cancer Carcinogenesis in Japanese Patients

**DOI:** 10.7759/cureus.48068

**Published:** 2023-10-31

**Authors:** Putri Damayanti, Sa Tin Myo Hlaing, Khine Zin Aung, Hiromasa Tsukino, Takuji Hinoura, Yoshiki Kuroda

**Affiliations:** 1 Department of Public Health, Faculty of Medicine, University of Miyazaki, Miyazaki, JPN; 2 Department of Urology, Junwakai Memorial Hospital, Miyazaki, JPN

**Keywords:** -318 c/t polymorphism, ctla-4, japanese, urothelial cancer, immunoregulatory molecule

## Abstract

Background

Urothelial cancer is one of the most common types of urinary system cancer and there are several factors that can influence its growth. One of the most prominent factors among these is genetics. The Cytotoxic T-Lymphocyte Antigen-4 (CTLA-4) gene is suspected to be a susceptibility gene in urothelial carcinoma. The aim of this study is to investigate polymorphism in the CTLA-4 gene (CTLA-4 -318 C/T) and whether it is associated with urothelial cancer.

Methods

The study population consisted of 253 cases and 272 controls. In this case-control study, DNA was extracted from peripheral blood cells, and the CTLA-4 -318C/T genotypes were detected using polymerase chain reaction-restriction fragment length polymorphism.

Results

C/T (adjusted OR (aOR) 3.37; 95%CI: 1.98-5.74) genotype, C/T + T/T (aOR 3.25; 95%CI: 1.96-5.39) genotype, and T allele (aOR 2.94 95%CI: 1.87-4.62) all indicated they are significant risk factors for urothelial cancer, with the effects of polymorphism being higher in the nonsmoker group than in the smoker group. Furthermore, the association between polymorphism and urothelial cancer carcinogenesis was similar among men and women.

Conclusions

This is the first study examining the association between CTLA-4 -318C/T polymorphism and urothelial carcinoma in Japanese patients. A significant association between CTLA-4 -318C/T polymorphism and urothelial cancer among Japanese patients was detected in this study. This supports the development of research on polymorphisms in urothelial cancer and is an important root of immunoreactions in cancer. We believe this study will be beneficial to clarify the relationship between CTLA-4 polymorphism and urothelial cancer.

## Introduction

Urothelial cancer is becoming one of the most lethal cancers globally. In 2020, 573,278 new cases and 212,536 deaths related to it were reported worldwide [1]. It is the fourth most common type of tumor in men, with the ratio between males and females being 3:1 [2]. The average age of diagnosis is 73 years [3]. In Japan, urothelial cancer is the eighth most prevalent cancer in males and the 17th most prevalent cancer in females. Urothelial cancer is defined as a cancer that arises from the epithelial surface of urologic organs, such as the kidney, ureter, bladder, and urethra [4]. Bladder cancer is the most prevalent such cancer, followed by upper tract urothelial carcinoma [5].

The carcinogenic features of urothelial cancer are thought to be multifactorial. The primary provoking factors are smoking, genetics, lifestyle, and environment. Smoking is the highest risk factor for urothelial cancer, followed by occupational exposure to carcinogenic agents, diet, and environmental pollution. An increasing number of recent studies have shown that genetic predisposition also has a significant impact on carcinogenesis [6].

The role of Cytotoxic T-Lymphocyte Antigen-4 (CTLA-4) was thought to be to provide an inhibitory effect on the antitumor immune response mediated by antigen-presenting cell (APC) interactions in retaining peripheral tolerance [7,8]. The attachment of the T cell receptor and the antigen-bound major histocompatibility complex on APCs is required to activate the T cell [9]. CTLA-4 is a CD28 homolog that acts as an inhibitory receptor for B7 [7,10]. CTLA-4 inhibition improves both the immune response [11] and tumor cell rejection [12].

CTLA-4 is located on chromosome 2q33 and consists of four exons in humans [13,14]. There are several CTLA-4 single nucleotide polymorphisms (SNPs) that play crucial roles in numerous autoimmune disorders and cancers [15]. Recent research suggests the CTLA-4 gene SNPs -318C/T could affect gene expression and alter the susceptibility to many kinds of cancer [16], such as cervical cancer [17-19], lymphoma [20], leukemia [21], and breast cancer [22]. Previous studies in the Chinese population showed a significant relationship between CTLA-4 +49A/G and bladder cancer [23,24] and a significant relationship between CTLA-4 CT60 and renal cell cancer in the Spanish population [25]. There have been no studies thus far concerning the association between this polymorphism and urothelial carcinoma patients in the Japanese population. This CTLA-4 polymorphism study may therefore provide useful insights and suggestions for specific immunotherapy applications for cancer. The aim of this study is to investigate the relationship between CTLA-4 -318 C/T polymorphism and urothelial cancer in Japanese patients.

## Materials and methods

Study subjects

The present study is a case-control study including a total of 253 urothelial cancer cases (bladder cancer: 221 patients, ureteral cancer: 16 patients, bladder and ureteral cancer: 16 patients) and 272 clinical controls from among the Japanese population. The urothelial cancer patients had been diagnosed at the University of Occupational and Environmental Health (UOEH) Hospital and the University of Miyazaki Hospital in Japan. Controls with noncancerous diseases were also recruited at the same hospitals around the same time.

All subjects were surveyed using a self-administered questionnaire that collected information on their sex, age, illness, and smoking status (active smoker, ex-smoker, and nonsmoker). We separated smokers (active smokers and ex-smokers) and nonsmokers. Participants with a history of occupational exposure to radiation, heavy metals, or carcinogenic agents were excluded. All participants were informed about the nature of the study, and each provided written informed consent. The Ethics Committee of the Faculty of Medicine, University of Miyazaki, approved this study (approval number: 239).

Genotyping

Each subject provided peripheral blood samples, and genomic DNA was extracted using a DNA extractor WB Kit (Wako Pure Chemical Industries, Osaka, Japan). A polymerase chain reaction (PCR)-restriction fragment length polymorphism was used to identify the C/T allele polymorphism in the CTLA-4 gene promoter region position -318 on chromosome 2q33 (rs5742909). The -318C/T polymorphism was amplified in a 247 bp product with primer 5’-AATGAATTGGACTGGATGG-3’ (forward) and 5’-TTACGAGAAAGGAAGCCGTG-3' (reverse). The PCR was conducted using a KAPA Taq EXtra PCR Kit (Nippon Genetics Co., Ltd., Tokyo, Japan). The DNA was denaturated at 94^o^C for two minutes, followed by 40 cycles of 94^o^C for 30 seconds, 60^o^C for 30 seconds, 72^o^C for 30 seconds, and a final extension at 72^o^C for six minutes. The PCR product was digested by the MseI restriction enzyme. The C/C genotype showed a single band of 226 bp, the C/T genotype showed three bands of 226 bp, 130 bp, and 96 bp, and the T/T genotype showed two bands of 130 bp and 96 bp.

Statistical analysis

The continuous variable is presented with a standard deviation and analyzed using a t-test. For categorical variable comparisons and assessing the potential for Hardy-Weinberg equilibrium, Pearson’s chi-squared test (χ2) was utilized. Multiple logistic regression was used in the multivariate analyses. To reduce the effect of confounding factors, age, sex, and smoking status were adjusted in the study. Statistically significant values were considered to be those with p < 0.05. All statistical analyses were performed using JMP® Pro 16 (2021; SAS Institute Inc., Cary, North Carolina, United States).

## Results

Table 1 shows the general characteristics regarding sex, age, and smoking history. The percentage of women in the control group was significantly more than the cases group. The mean age was 69.5 years (SD 11.0) for the cases group and 68.3 years (SD 12.6) for the control group. There were significantly more smokers in the cases group (56.9%) than in the control group (43.1%). However, there was no difference in smoking status among cases and controls, compared by sex. This means the cases group contained many more men than the control group.

**Table 1:** The characteristics of cases and controls ^*^ P < 0.05; ^** ^P < 0.01

	Cases (males/females)	Controls (males/females)
n	253 (207/46)	272 (131/141)**
Age (years) (Mean±SD)	69.5±11.0 (69.4±10.9/69.7±11.8)	68.3±12.6 (67.0±12.59/69.5±12.4)
Nonsmoker	72 (35/37)	135 (22/113)
Smoker	181(172/9)	137 (109/28)**

Table 2 presents the distribution of CTLA-4 -318C/T (rs5742909) genotypes among both the cases and control groups. The genotype distribution for CTLA-4 -318C/T polymorphisms in the control group was on the Hardy-Weinberg equilibrium (P = 0.146). The number of C/T and C/T+T/T genotypes was significantly higher in the cases group than in the control group. The adjusted ORs (aOR) were 3.37 (95%CI: 1.98-5.74) and 3.25 (95%CI: 1.96-5.39), respectively.

**Table 2:** The distribution of genotypes among cases and controls ^#^Adjusted by age, sex, and smoking status; ^*^ P < 0.05; ^**^ P < 0.01

	Genotype	Cases (n=253)	Controls (n=272)	OR (95% CI)	Adjusted OR (95% CI)^#^
-318C/T	C/C (%)	187 (73.9)	236 (86.8)	Ref	Ref
	C/T (%)	58 (22.9)	33 (12.1)	2.21 (1.39–3.54)**	3.37 (1.98–5.74)**
	T/T (%)	8 (3.2)	3 (1.1)	3.37 (0.88–12.86)	2.38 (0.59–9.60)
	C/T + T/T (%)	66 (26.1)	36 (13.2)	2.31 (1.48–3.62)**	3.25 (1.96–5.39)**
	C allele	432 (85.4)	505 (92.8)	Ref	Ref
	T allele	74 (14.6)	39 (7.2)	2.22 (1.47–3.34)**	2.94 (1.87–4.62)**

We evaluated the relationship between the carcinogenesis of urothelial cancer and the CTLA-4 -318C/T (rs5742909) genotypes, stratified by smoking status and sex, as shown in Tables 3-4. The results indicate the C/T and C/T + T/T genotypes were more significant in the cases than in the control group. Also in the nonsmoker group, the C/T + T/T genotype was significantly more in the cases (Table 3) compared to the smoker group. In addition, the C/T and C/T + T/T genotypes were more significant in the cases group than the control group in both males and females (Table 4).

**Table 3:** The distribution of genotypes among cases and controls according to smoking status ^#^ Adjusted by age and sex; ^*^ *P *< 0.05;  ^**^ *P *< 0.01

	Genotype	Cases(n=72)	Controls(n=135)	Crude OR (95% CI)	Adjusted OR (95% CI)^#^
Nonsmoker	C/C (%)	42 (58.3)	115 (85.2)	Ref	Ref
	C/T (%)	28 (38.9)	18 (13.3)	4.26 (2.14–8.49)**	6.10 (2.83–13.18)**
	T/T (%)	2 (2.8)	2 (1.5)	2.73 (0.37–20.06)	1.19 (0.13–10.39)
	C/T + T/T (%)	30 (41.7)	20 (14.8)	4.11 (2.11–8.00)**	5.31 (2.54–11.10)**
	C allele	112 (77.8)	248 (91.9)	Ref	Ref
	T allele	32 (22.2)	22 (8.1)	3.22 (1.79–5.79)**	3.57 (1.90–6.69)**
	Genotype	Cases(n=181)	Controls(n=137)	Crude OR (95% CI)	Adjusted OR (95% CI)^#^
Smoker	C/C (%)	145 (80.1)	121 (88.3)	Ref	Ref
	C/T (%)	30 (16.6)	15 (10.9)	1.67 (0.86–3.25)	1.91 (0.95–3.85)
	T/T (%)	6 (3.3)	1 (0.7)	5.01 (0.59–42.16)	4.16 (0.49–35.21)
	C/T + T/T (%)	36 (19.9)	16 (11.7)	1.88 (0.99–3.55)	2.08 (1.07–4.06)*
	C allele	320 (88.4)	257 (93.8)	Ref	Ref
	T allele	42 (11.6)	17 (6.20)	1.98 (1.10–3.57)**	2.12 (1.15–3.89)**

**Table 4:** The distribution of genotypes among cases and controls according to sex ^#^ Adjusted by age and smoking status ^*^ P < 0.05;^ **^ P < 0.01

	Genotype	Cases(n=207)	Controls(n=131)	Crude OR (95% CI)	Adjusted OR (95% CI)^#^
Males	C/C (%)	159 (76.8)	120 (91.6)	Ref	Ref
	C/T (%)	40 (19.3)	9 (6.9)	3.33 (1.56–7.14)**	3.59 (1.66–7.73)**
	T/T (%)	8 (3.9)	2 (1.5)	3.00 (0.63–14.38)	2.99 (0.62–14.54)
	C/T + T/T (%)	48 (23.2)	11 (8.4)	3.27 (1.63–6.57)**	3.48 (1.72–7.03)**
	C allele	358 (86.5)	249 (95.0)	Ref	Ref
	T allele	56 (13.5)	13 (5.0)	2.03 (1.32–3.12)**	2.41 (1.51–3.84)**
	Genotype	Cases(n=46)	Controls(n=141)	Crude OR (95% CI)	Adjusted OR (95% CI)^#^
Females	C/C (%)	28 (60.9)	116 (82.3)	Ref	Ref
	C/T (%)	18 (39.1)	24 (17.0)	3.11 (1.49–6.50)**	3.12 (1.49–6.54)**
	T/T (%)	0 (0)	1 (0.7)	–	–
	C/T + T/T (%)	18 (39.1)	25 (17.7)	2.98 (1.43–6.21)**	3.00 (1.43–6.26)**
	C allele	74 (80.4)	256 (90.8)	Ref	Ref
	T allele	18 (19.6)	26 (9.2)	2.40 (1.25–4.61)**	2.40 (1.25–4.62)**

## Discussion

In this study, we investigated the relationship between the CTLA-4 -318C/T polymorphism and the carcinogenesis of urothelial cancer among Japanese patients. Our results indicated the frequency of C/T and C/T+T/T genotypes were higher in the cases than the controls. We obtained similar results with evaluations separated by both sex and smoking status. Therefore, it appears the T allele could be a risk factor for urothelial cancer.

Considering CTLA-4 plays a primary role in the negative regulation of T-cell proliferation and activation [26], if there is any disturbance in the CTLA-4 expression or function, the T-cell proliferation and activation could be disrupted. CTLA-4 polymorphism contributes to the development of autoimmune diseases [15,27,28] and malignancies, such as renal and breast cancers [25,29]. Since the function of the promoter region is the expression of the CTLA-4 protein on the cell surface, if there is a C/T polymorphism in the -318 position, the gene expression will likely change [30,31]. Besides urothelial cancer, previous studies also indicate there is a relationship between CTLA-4 -318 C/T polymorphism in cervical cancer [17-19,32], leukemia [21], melanoma [33], thymoma [34], lymphoma [20], skin cancer [35], and breast cancer [22]. Therefore, we evaluated the relationship between the CTLA-4 -318C/T polymorphism and urothelial cancer carcinogenesis.

We also investigated the association between urothelial cancer and CTLA-4 -318C/T polymorphism, as stratified by smoking status and sex. We found C/T and C/T+T/T were predominant in urothelial cancer compared to the controls, regardless of smoking status and sex. The T allele was far more present in the urothelial cancer cases than in the controls. 

The aOR was higher in nonsmokers than in the smoker group. It is well-established that tobacco smoke contains over 60 carcinogenic substances that can induce DNA damage, mutations, changes in DNA methylation, and lead to SNPs in genome and sequences that are linked to various types of cancer [36-38], including urothelial cancer [39-41]. Hect et al. conducted research on the genomic and bioinformatic approaches for the association of cancer and smoking, claiming most genes were damaged by smoking, whereas one gene did not show any health risk but still had the potential for tobacco-specific nitrosamine production and cancer development [36]. Therefore, although smoking can certainly be carcinogenic, the effect of polymorphism could be relatively weak. For example, Kuroda et al. have indicated the pro allele of the p53 codon 72 polymorphism increases in urothelial cancer cases in lighter smokers [42], whereas Khoury et al. [43] and Wang et al. [44] also reported genetic differences in cancer risk might be smaller with high loads of carcinogen exposure. Their results support our own.

On the other hand, since the OR of C/T+T/T in males was similar to that in females, the relationship between urothelial cancer and CTLA-4 -318C/T polymorphism was not different between males and females. However, the rationale for explaining this different incidence is unclear [45].

Results concerning the interaction between gene polymorphisms and gender were contradictory. It has been reported that TAP1 polymorphism has increased susceptibility in both genders in colon cancers [46]. Holipah et al. reported that mutant type (G/G) of PER1 (rs3027188) is protective against colon cancer in women [47] and indicated a mutant T/T genotype of PER3 rs2640908 became a protective factor against colon cancer in men [48]. Dresler et al. reported the polymorphisms of cytochrome P450 1A1 (CYP1A1) (exon 7) were a particular risk factor for lung cancer in women, claiming the different effects of polymorphism by gender were derived from the influence of female sex steroid hormones such as estrogen [49]. However, the details have not been elucidated in the literature at present. Further study will be needed.

There are some limitations to this research. First, we do not have information about other potential confounding factors as a cause of cancer such as alcohol consumption, smoking period, and number of cigarettes. Our study does not assess the tumor stage, location, pathological type, and prognosis. Furthermore, since we were required to adopt the hospital controls, we cannot rule out the possibility that our controls may contain some kind of bias. Despite these limitations, we believe this study could be beneficial in better understanding the relationship between CTLA-4 polymorphism and urothelial cancer.

## Conclusions

This is the first study examining the association between CTLA-4 -318C/T polymorphism and urothelial carcinoma in Japanese patients. A significant association between CTLA-4 -318C/T polymorphism and urothelial cancer was detected in this study. This supports the development of research on polymorphisms in urothelial cancer and is an important root of immunoreactions in cancer. We believe this study could be beneficial in clarifying the relationship between CTLA-4 polymorphism and urothelial cancer.
